# Differences in somatosensory processing due to dominant hemispheric motor impairment in cerebral palsy

**DOI:** 10.1186/1471-2202-15-10

**Published:** 2014-01-11

**Authors:** Inmaculada Riquelme, Iván Padrón, Ignasi Cifre, Ana M González-Roldán, Pedro Montoya

**Affiliations:** 1 Research Institute of Health Sciences (IUNICS), University of the Balearic Islands, Carretera de Valldemossa km 7.5, Palma 07122, Spain; 2 Department of Nursing and Physiotherapy, University of the Balearic Islands, Palma, Spain; 3 Department of Developmental Psychology and Education, University of La Laguna, La Laguna, Spain

**Keywords:** Somatosensory processing, Cerebral palsy, Motor impairment, Hemispheric asymmetry

## Abstract

**Background:**

Although cerebral palsy (CP) is usually defined as a group of permanent motor disorders due to non-progressive disturbances in the developing fetal or infant brain, recent research has shown that CP individuals are also characterized by altered somatosensory perception, increased pain and abnormal activation of cortical somatosensory areas. The present study was aimed to examine hemispheric differences on somatosensory brain processing in individuals with bilateral CP and lateralized motor impairments compared with healthy controls. Nine CP individuals with left-dominant motor impairments (LMI) (age range 5–28 yrs), nine CP individuals with right-dominant motor impairments (RMI) (age range 7–29 yrs), and 12 healthy controls (age range 5–30 yrs) participated in the study. Proprioception, touch and pain thresholds, as well as somatosensory evoked potentials (SEP) elicited by tactile stimulation of right and left lips and thumbs were compared.

**Results:**

Pain sensitivity was higher, and lip stimulation elicited greater beta power and more symmetrical SEP amplitudes in individuals with CP than in healthy controls. In addition, although there was no significant differences between individuals with RMI and LMI on pain or touch sensitivity, lip and thumb stimulation elicited smaller beta power and more symmetrical SEP amplitudes in individuals with LMI than with RMI.

**Conclusions:**

Our data revealed that brain processing of somatosensory stimulation was abnormal in CP individuals. Moreover, this processing was different depending if they presented right- or left-dominant motor impairments, suggesting that different mechanisms of sensorimotor reorganization should be involved in CP depending on dominant side of motor impairment.

## Background

Cerebral palsy (CP) is defined as a group of permanent motor disorders that are attributed to non-progressive disturbances in the developing fetal or infant brain. Nevertheless, it has been recently shown that CP could be associated with somatosensory alterations, including abnormal perception of touch and altered pain sensitivity [1]. Indeed, research on somatosensory processing has revealed that individuals with CP are characterized by poor tactile discrimination, stereognosis and proprioception [2-4], as well as increased pain [5] and abnormal activation of cortical somatosensory areas [6]. Moreover, somatosensory parameters have been associated with clinical measures of motor impairment in persons with CP and other neurological pathologies such as multiple sclerosis, spinal cord injuries and cerebrovascular accidents [7-11]. Neuroimaging studies have also provided evidence of significant alterations in white matter fibers connecting to somatosensory cortex, suggesting that CP injuries reflect a disruption of sensory as well as motor pathways [12-14].

Although neurophysiological mechanisms involved in altered processing of bodily information in CP are still unknown, there is evidence of an abnormal sensorimotor integration in hemiplegic CP [15], as it occurs in other movement disorders such as Parkinson’ disease, Huntington’s disease, dystonia, and tics [16-18]. Furthermore, it is possible that motor reorganization in children with congenital hemiplegic CP occurs by preserving motor representations of the affected arm in the intact hemisphere. In this sense, studies with transcranial magnetic stimulation (TMS) have provided evidence of ipsilateral corticospinal projections from the undamaged motor cortex to the affected hand [15,19-24]. Moreover, it has been proposed that somatosensory deficits could be due to secondary effects provoked by motor limitation [25]. Thus, reduced and stereotypical pattern of spontaneous movements in patients with hemiplegic CP would result in abnormal sensory feedback and altered cortical reorganization, thus leading to asymmetric somatosensory processing deficits [25,26]. A case study by Ragazzoni and colleagues (2002) [27] has further showed that somatosensory function of the affected (right) arm was preserved, whereas motor function was poor despite fast-conducting ipsilateral cortico-motoneuronal output from primary motor cortex of the intact hemisphere to the affected arm. This finding seems to suggest that different forms of motor and somatosensory reorganization are involved in congenital brain injury, and that fast-conducting connections between primary cortex areas and ipsilateral spinal cord are not sufficient for preservation or recovery of function.

In the present study, we examined the effects of lateralized motor impairment on somatosensory brain processing by using somatosensory evoked potentials (SEP) in persons with bilateral CP. For this purpose, we divided our sample into CP individuals with either right-dominant dominant side of motor impairment (RMI) or CP left-dominant side of motor impairment (LMI) to test possible differences on propioception, touch and pain sensitivity, as well as early SEP components elicited by non-painful stimulation of lips and thumbs at each dominant side of motor impairment. Furthermore, we compared these CP individuals with a group of healthy volunteers to quantify the impairments in somatosensory processing due to cerebral palsy.

## Methods

### Participants

Thirty individuals with bilateral cerebral palsy (CP) were recruited from educational and occupational centers established in the island of Majorca (Spain), and invited to participate in the study. Patients were classified according with the criteria of the Surveillance of Cerebral Palsy in Europe (SCPE, 2000) into the following categories: bilateral spastic (including diplegia and quadriplegia), dyskinetic and ataxic. The diagnosis of spastic hemiplegia was specifically excluded. The hand motor function of each participant was assessed by using the House Functional Classification [28]. Those CP individuals with no clear asymmetric motor performance were also excluded from the present study. Eighteen participants showing different asymmetric motor performance were selected on the basis of their scores for both hands in the House Functional Classification (Table 1). The existence of an asymmetrical brain damage in neuroimaging explorations was only confirmed in 33% of the cases by checking their health records.

**Table 1:** Clinical characteristics of individuals with cerebral palsy (R = right, L = left M = male, F = female, BS = bilateral spastic, D = dyskinetic and A = ataxic)

***Dominant motor impairment***	***HOUSE right hand***	***HOUSE left hand***	***CP subgroup***	***Gestational age *** **( *weeks *)**	***GMFCS***	***Mental retardation***	***Epilepsy***	***Medication***	***Neuroimaging findings***
R	2	6	BS	36	2	No	Yes	Antiepileptic	Ventricular asymmetry in supratentorial area, wide ventricular cavities
R	5	7	A	40	4	No	No	No	Cortical atrophy with asymmetrical subcortical damage in temporal lobes, brainstem atrophy and hypoplasia
R	6	8	A	40	1	Moderate	No	No	Collapsed lateral ventriculi, periencephalic cavity in occipital and posterior parietal areas
R	3	6	BS	28	2	No	No	No	N/A
R	3	6	BS	40	1	No	No	No	N/A
R	6	8	BS	40	1	Severe	No	Antidepressants	N/A
R	1	4	A	40	2	Severe	Yes	No	N/A
R	6	8	BS	40	1	Moderate	No	No	N/A
R	3	6	BS	32	2	No	No	No	N/A
L	4	1	BS	20	4	Mild	Yes	Antiepileptic	N/A
L	7	5	BS	24	1	No	No	Musc. relaxant	Asymmetry in Rolando cortex with periventricular cyst in left hemisphere
L	7	5	BS	40	2	Moderate	Yes	Antiepileptic	Corpus callosum and formix hypoplasia, septum pellucidum cyst, diffuse cortical atrophy, abnormal EEG activity in left parietal lobe
L	7	5	BS	41	3	No	No	No	N/A
L	8	6	BS	31	3	Mild	No	Antidepressants	Leukomalacia with dilatation of left temporal lobe
L	8	5	BS	40	3	No	Yes	Antiepileptic	N/A
L	8	6	BS	40	1	Moderate	No	No	N/A
L	4	0	BS	40	3	Moderate	No	No	N/A
L	6	3	D	42	5	No	Yes	Antidepressants	N/A

Individuals with CP were classified according to the dominant side of motor impairments into two groups: 1) nine CP participants with dominant right-sided motor impairment (RMI) (3 females; mean age: 18y 3mo, range 5-28y), and 2) nine CP with dominant left-sided motor impairment (LMI) (3 females; mean age: 15y 4mo, range 7-29y). Table 1 displays clinical characteristics for each group of participants. Subjects or their parents reported their age and sex. Type of cerebral palsy, gestational age, cognitive level, presence of epilepsy and medication were obtained from participant’s health records. The level of motor impairment was also determined by using the Gross Motor Function Classification Scale (GMFCS) [29].

In addition, 12 right-handed healthy volunteers (3 females; mean age: 18y 1mo, range 5-30y) were recruited from educational centers and took part in the study.

All participants granted written informed consent according with the Declaration of Helsinki. In the case of children, parents were informed and their written consent was obtained. The study was approved by the Ethics Committee of the Regional Government of the Balearic Islands (Spain).

### Somatosensory assessment

Behavioral measures of somatosensory processing and sensitivity were obtained by using following tasks:

#### Proprioception

Proprioceptive skills were assessed by asking participants to perform unilateral movements of the upper limb with eyes closed and to perform the same movement with the contralateral limb. Each single movement was repeated five times and the average number of correct trials (defined as a movement with less than 10 degrees of difference with respect to the final position of the target limb) was used as an index of proprioceptive skills. This procedure has been used successfully in previous studies [2].

#### Touch

Touch sensitivity (expressed in g/mm^2^) was measured bilaterally at two body locations (lips and thumb finger) by using von Frey monofilaments [30] with different diameters (0.14-1.01 mm). The test was performed by touching the skin in a perpendicular way with the monofilament, pressing it slowly down until it buckles, holding it steady during 1.5 seconds, and removing it in the same way as it was applied. After several trials to assure the understanding of the procedure, subjects were instructed to notify the experimenter if they felt any sensation of touch by saying “yes” or “no”. The procedure started with a thick filament and depending on subjects’ answers, thicker or thinner filaments were applied. The sensitivity score for each body location was calculated as the mean of the three thinnest filaments detected. Null stimuli were also used to find false positive responses and responses delayed more than 3 seconds were noted as abnormal. Body locations were stimulated in a pseudo-randomized order.

#### Pressure pain

Pressure pain thresholds (expressed in kgf/cm^2^) were measured with a digital dynamometer and using a flat rubber tip (1 cm^2^). Subjects were asked to say ‘pain’ or to make a significant gesture when the pressure became painful. Pressure was released when either pain detection threshold or maximum pressure of the algometer (13.0 kgf/cm^2^) was reached. Pressure stimuli were applied bilaterally in pseudo-randomized order at two body locations (lips and thumb finger). Subjects were previously familiarized with the procedure by using non-painful ranges to relieve potential anxiety. The reliability of this procedure for assessing pain sensitivity has been demonstrated in previous studies [31].

### EEG recording and data processing

Somatosensory evoked-potentials (SEP) elicited by tactile stimulation of four body locations (right lip, left lip, right hand and left hand) were recorded in a sound attenuated, semi-darkened room. Subjects were seated in a reclining chair and encouraged to relax and to keep their eyes closed. In younger children, mother was allowed to remain in the room if the child was anxious. Non-painful tactile computer-controlled impulses were delivered bilaterally at the body locations by using a pneumatic stimulator, consisting of a small membrane attached to the body surface by a plastic clip and fixated with adhesive strips. Each stimulation block consisted of 120 stimuli of 100 ms duration with an approximate pressure of 2 bars and a variable inter-stimulus interval of 1000 ± 50 ms. The order of the stimulation blocks was counterbalanced across subjects. Similar tactile stimulation has been already used in previous research of our lab to study somatosensory processing in persons with CP [6]. Electrical brain activity was recorded by using a 20-channels EEG amplifier with electrodes located according with the international 10/20 system and referenced to Cz. Vertical electrooculograms (EOG) were recorded bipolarly from the outer canthi of both eyes. Electrode impedance was kept below 10 kOhm. Sampling rate was set at 1000 Hz and filter bandpass at 0.1-40 Hz. A digital signal from the tactile stimulation device was used as a trigger for SEP acquisition. SEPs were averaged relative to a 100-ms prestimulus baseline. Eye movement artifacts were corrected by using Gratton & Coles algorithm [32]. An artifact rejection protocol was applied with following criteria: 75 μV as maximal allowed voltage step/sampling point, ±75 μV as minimum and maximum allowed amplitudes, and 75 μV as maximum allowed absolute difference. Individual averages were obtained for each body location and electrode location. One subject of the RMI group had to be eliminated from all analyses because their EEG recordings did not meet specified criteria. In addition, three subjects from the RMI group and one from the LMI were eliminated from those analyses involving either stimulation of thumbs (two subjects) or lips (two subjects) due to excessive artifacts in those conditions.

Within the first 150-ms interval, SEPs elicited by non-painful tactile stimuli are usually characterized by a prominent positive peak around 50 ms (P50), followed by a second positive peak around 100 ms after stimulus onset (P100) [6,33,34]. Although both peaks were clearly observable after thumb stimulation in our grand averages, peak detection was difficult in CP individual SEP averages and, therefore, mean amplitudes were computed in two time-windows: 20–70 ms and 70–120 ms after stimulus onset. Moreover, event-related brain oscillations elicited by somatosensory stimuli were analyzed by computing the relative increases or decreases of each frequency power with respect to the baseline interval (100 ms before stimulus onset). Time-frequency analyses of evoked power were computed by using a Mortlet wavelet (width 7 cycles) by convolution in the frequency domain on single trials in the time-windows 20–70 ms and 70–120 ms after stimulus onset. An average absolute power value was calculated separately for each electrode and following frequency bands: theta (4–8 Hz), alpha (8–12 Hz), and beta (12–20 Hz).

### Statistical analyses

Differences on somatosensory processing were analyzed by comparing CP individuals, as a whole, with healthy controls, as well as by comparing CP individuals grouped according to the dominant side of motor impairments (LMI vs. RMI). Group differences on proprioception measures were computed with non-parametrical mean comparisons. Differences on touch and pain thresholds were tested by using analyses of variance (ANOVAs) for repeated measures with a between-subject factor GROUP and a within-subject factor BODY SIDE (stimulation at left vs. right body side). SEP amplitudes and frequency power spectra of somatosensory-evoked oscillations over centro-parietal electrodes (C3, C4, P3, P4) were analyzed by ANOVA with the between-subject factor GROUP, as well as the within-subject factors BODY SIDE and HEMISPHERE (contralateral vs. ipsilateral to stimulation side). For all analyses, interaction effects were assessed by using post-hoc mean comparison tests provided by the ANOVA procedure in SPSS.

## Results

### Comparison between individuals with cerebral palsy (CP) and healthy controls

#### Somatosensory assessment

**Figure 1:**
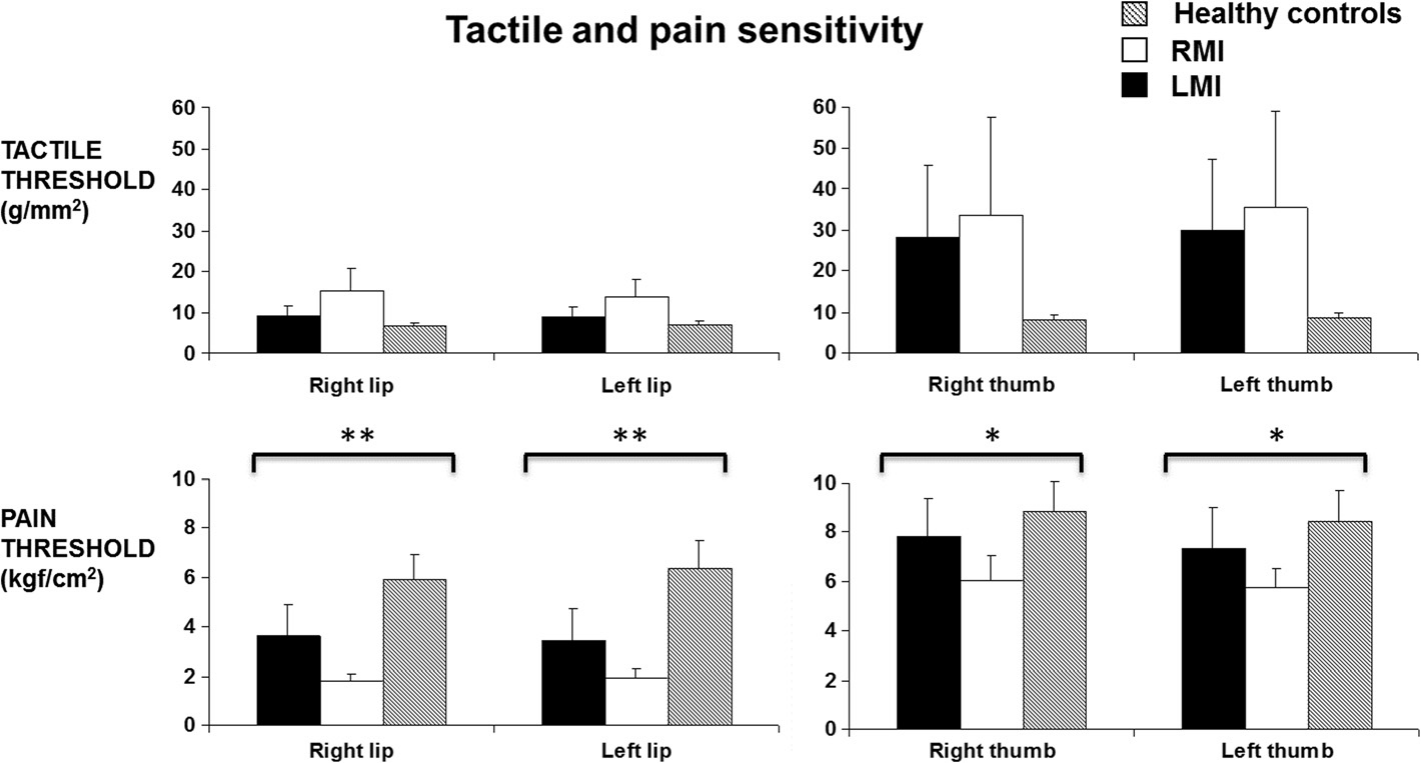
**Touch and pain sensitivity at lips and thumbs in healthy individuals and CP individuals with right- (RMI) and left-dominant motor impairments (LMI).**

Figure 1 displays mean scores of touch and pain sensitivity at the lip and thumb for healthy controls and CP individuals grouped by dominant side of motor impairment (right- [RMI] vs. left-sided motor impairment [LMI]). Statistical analyses revealed that healthy controls had higher pain thresholds than CP individuals in lips (F(1,26) = 21.7, p < .001) and in thumbs (F(1,25) = 8.1, p < .01). No significant group differences were found on touch sensitivity or proprioceptive skills.

#### Amplitude analyses of somatosensory evoked potentials

**Figure 2:**
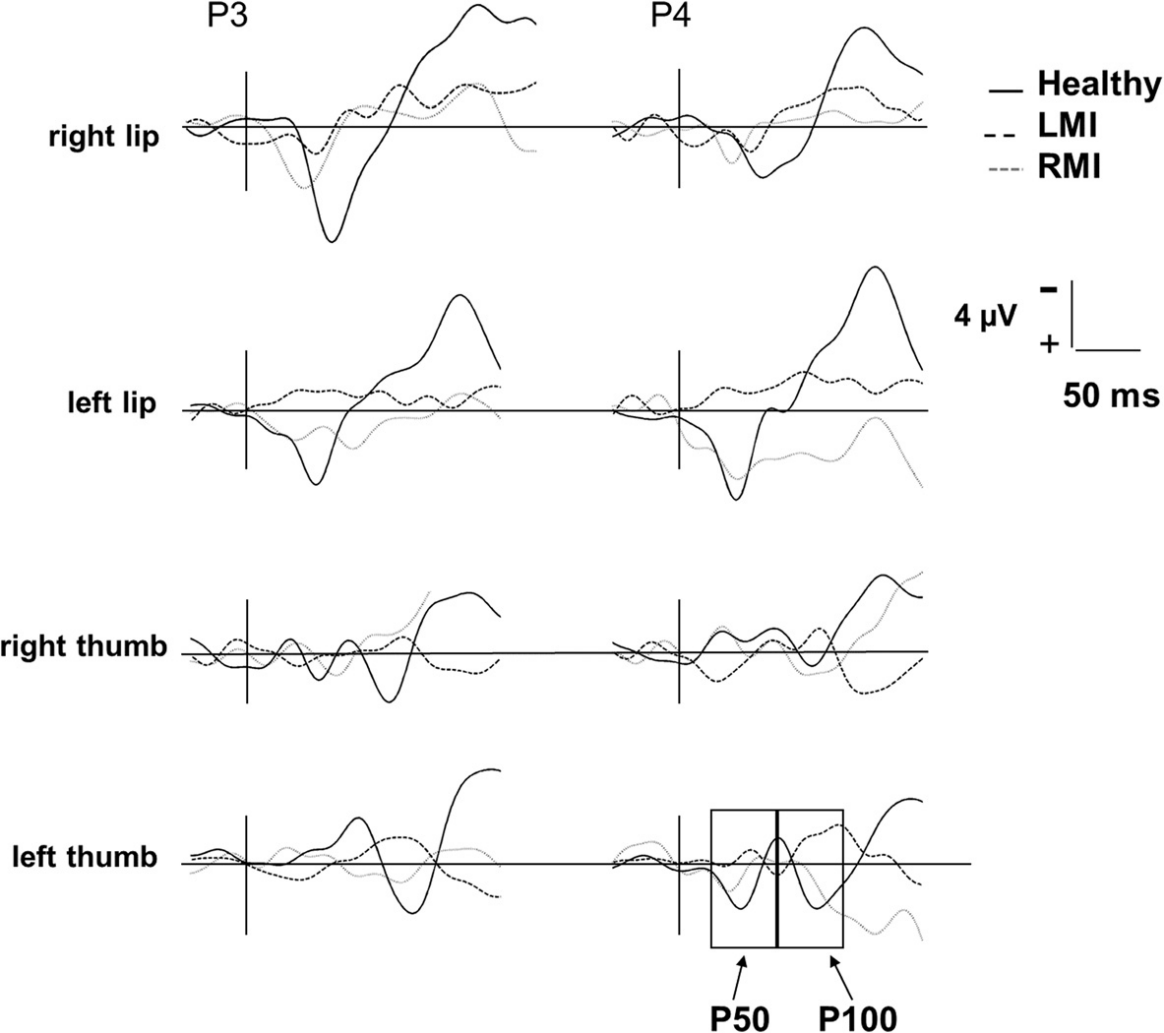
**Somatosensory evoked potentials (SEPs) elicited by stimulation of lips and thumbs over contralateral and ipsilateral hemispheres in healthy individuals, CP individuals with right- (RMI) and left-dominant motor impairments (LMI).**

Somatosensory evoked potentials (SEPs) elicited by stimulation of lips and thumbs were characterized in healthy controls by a positive peak between 20 and 70 ms after stimulus onset (P50), which was followed by a second positive deflection between 70 and 120 ms (P100). The scalp topography of both components in healthy controls indicated that they were more prominent over centro-parietal and parietal regions of the hemisphere contralateral to the stimulation side than over the ipsilateral hemisphere (Figure 2).

For lip stimulation, a significant GROUP x BODY SIDE x HEMISPHERE effect (F(1,25) = 4.5, p < .05) was yielded on mean SEP amplitudes in the time-window 20–70 ms. Post-hoc mean comparisons showed an asymmetrical somatosensory processing in healthy controls (higher amplitudes over the right than over the left hemisphere when left lip was stimulated) (p < .05), whereas no brain asymmetries were observed in CP individuals. However, SEP amplitudes in the time-window 70–120 ms were overall higher over the contralateral than over the ipsilateral hemisphere (F(1,25) = 11.0, p < .01).

For thumb stimulation, no significant effects were observed on SEP amplitudes in none of the two time-windows (20–70 ms and 70–120 ms).

#### Time-frequency analyses of somatosensory evoked oscillations

**Figure 3:**
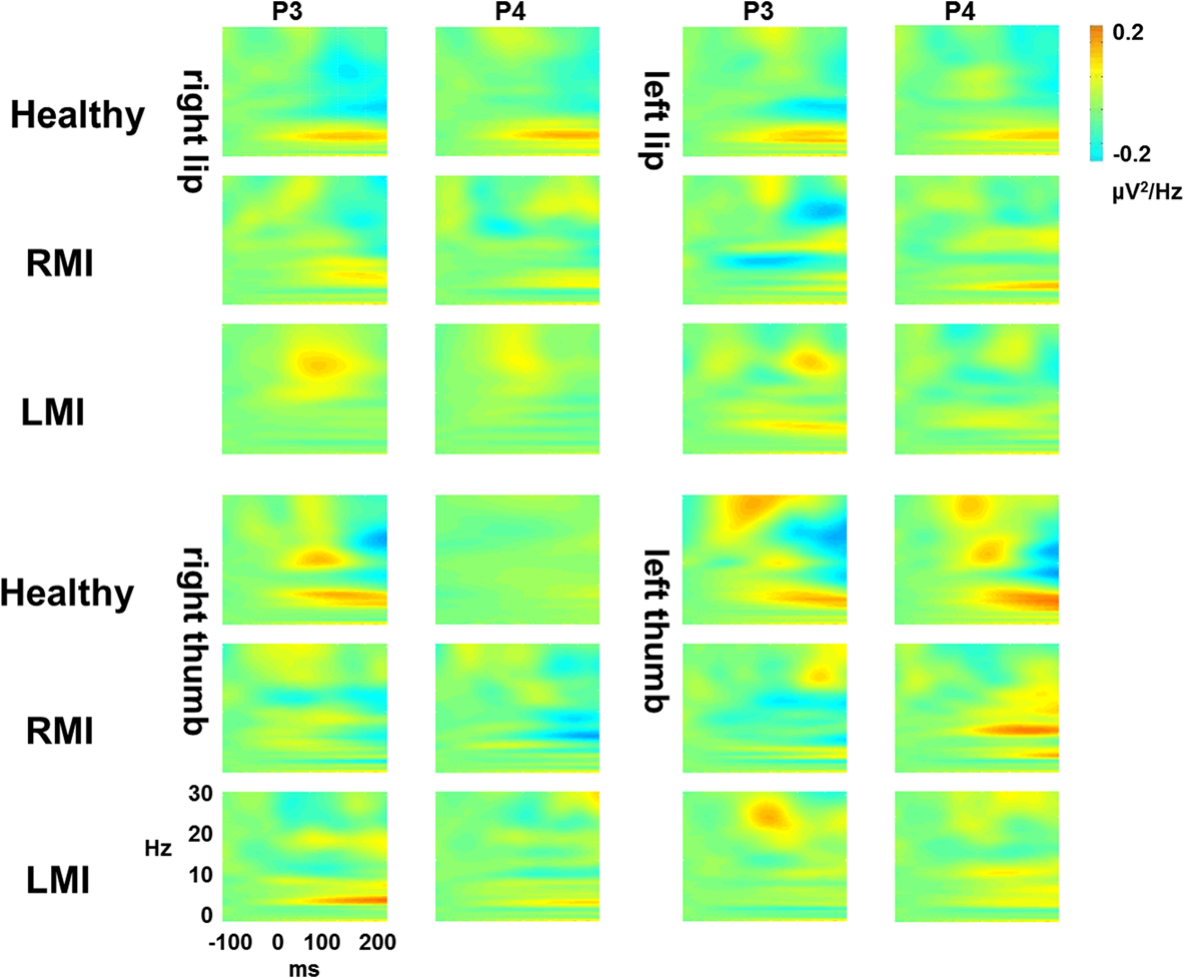
**Time-frequency power of somatosensory evoked oscillations elicited by stimulation of lips and thumbs over contralateral and ipsilateral hemispheres in healthy individuals and CP individuals with right- (RMI) and left-dominant motor impairments (LMI).**

Temporal changes in power spectra of the somatosensory evoked oscillations elicited by stimulation of lips and thumbs are shown in Figure 3 over the contralateral and ipsilateral hemispheres.

For lip stimulation, no significant effects were found on theta, alpha, and beta band power in the time-window 20–70. Nevertheless, CP individuals exhibited higher beta power than healthy controls in the time-window 70–120 ms (F(1,27) = 8.7, p < .01). Moreover, a significant GROUP x BODY SIDE interaction effect was found (F(1,27) = 9.0, p < .01), indicating that right lip stimulation elicited higher beta power in CP individuals than in healthy controls (p < .01), and that healthy controls displayed higher beta power when the left lip was stimulated as compared with the right lip (p < 05).

For thumb stimulation, a significant HEMISPHERE effect (F(1,27) = 4.9, p < .05) showed overall higher beta power over the contralateral than over the ipsilateral hemisphere in the time-window 20–70 ms. No significant effects were found on beta, alpha and theta band power in the time-window 70–120 ms.

### Comparisons between CP individuals with right- (RMI) vs. left-dominant motor impairments (LMI)

#### Somatosensory assessment

No significant effects of GROUP (RMI vs. LMI), BODY SIDE or GROUP x BODY SIDE were found on touch or pain sensitivity measures. No significant group differences were found on proprioceptive skills.

#### Amplitude analyses of somatosensory evoked potentials

For lip stimulation, CP individuals with RMI displayed higher SEP amplitudes than CP individuals with LMI in the time-window 20–70 ms (F(1,15) = 9.9, p < .01). Furthermore, a significant GROUP x HEMISPHERE interaction effect (F(1,15) = 4.9, p < .05) indicated that CP individuals with RMI yielded higher SEP amplitudes over the contralateral than over the ipsilateral hemisphere (p < .05), whereas no hemispheric differentiation appeared in CP individuals with LMI. In addition, a significant HEMISPHERE effect in the time-window 70–120 ms (F(1,15) = 4.7, p < .05) indicated higher SEP amplitudes over the contralateral than over the ipsilateral hemisphere in all CP participants.

For thumb stimulation, a significant GROUP effect revealed higher SEP amplitudes in RMI than in LMI individuals in the time-window 70–120 ms (F(1,14) = 7.8, p < .05). Moreover, a significant GROUP x BODY SIDE interaction effect was found (F(1,14) = 7.5, p < .05). Post-hoc mean comparisons revealed that right thumb stimulation elicited higher SEP amplitudes in RMI than in LMI individuals (p < .01), and that RMI individuals showed higher SEP amplitudes when right thumb was stimulated compared with left thumb stimulation (p < .05). No significant effects were observed on SEP amplitudes in the time-window 20–70 ms.

#### Time-frequency analyses of somatosensory evoked oscillations

For lip stimulation, no significant effects were found on beta, alpha and theta band power in none of the time-windows (20–70 ms and 70–120 ms).

For thumb stimulation, a significant GROUP x HEMISPHERE interaction effect was observed on beta band power in the time-window 20–70 ms (F(1,15) = 5.6, p < .05). Post-hoc comparisons indicated higher beta power over the contralateral than over the ipsilateral hemisphere in CP individuals with RMI (p < .05), whereas no significant differences were observed in CP individuals with LMI. No significant effects were found on beta, alpha and theta band power in the time-window 70–120 ms.

## Discussion

This study was aimed to evaluate the effects of lateralized motor impairment on somatosensory perception and brain processing of non-painful tactile stimulation in individuals with bilateral cerebral palsy (CP). Our findings revealed that although individuals with CP exhibited lower pain sensitivity than healthy controls, there were no significant differences on touch sensitivity, pain sensitivity or proprioception between CP individuals with right- (RMI) and left-dominant motor impairments (LMI). Regarding somatosensory brain processing, we found that lip stimulation elicited higher beta power, but more similar SEP amplitudes over the contra- and the ipsilateral hemispheres in CP individuals than in healthy controls. Moreover, lip and thumb stimulations elicited smaller and more symmetrical SEP amplitudes, as well as reduced beta power (only for thumb stimulation) in CP individuals with LMI than in CP individuals with RMI.

Thus, our results revealed an altered somatosensory processing in individuals with CP, as measured by SEP amplitudes and frequency power. The pattern of brain activation displayed by our individuals with CP after stimulation of thumbs and lips seems to be different from that observed in healthy controls. Reduced beta power and enhanced SEP amplitudes over somatosensory cortices have been observed in healthy controls following somatosensory stimulation, suggesting that these changes might be interpreted as an activation of cortical networks involved in somatosensory processing [35]. In the present study, healthy individuals showed a desynchronization in the beta frequency band in response to touch stimulation of lips and thumbs. In contrast, our individuals with CP appeared to display an increased beta power over the contralateral hemisphere, particularly in CP individuals with RMI. These findings are in agreement with previous studies showing that, although CP is mainly characterized by motor impairments, brain processing of incoming somatosensory information is also significantly altered in this pathology. Thus, for instance, neuroimaging research has found that children with periventricular leukomalacia show more severe injury in posterior white matter fibers connecting the thalamus to the sensory cortex than in descending corticospinal tracts [14]. Moreover, it has been demonstrated that deficits on somatosensory processing (reduced touch sensitivity, proprioception and strength) in CP could be related to injury severity of diffuse thalamocortical projections to somatosensory and parietal cortices [26]. In this sense, our results provide further empirical evidence for an abnormal brain processing of bodily information in CP individuals.

Motor function seems to be often asymmetric in CP, even in individuals with bilateral lesions [36-38]. This asymmetry has been reported by using physiological measures such as nerve conduction velocities or dichotic listening processing [39,40]. In the present study, we found significant differences on the pattern of hemispheric brain activation elicited by somatosensory stimulation depending on the dominant side of motor impairment. In this sense, participants with RMI displayed enhanced responses to bodily stimulation over contralateral as compared to ipsilateral hemispheres, whereas individuals with LMI showed no hemispheric differences. Moreover, CP subjects with RMI showed higher SEP amplitudes when the affected side of the body was stimulated, while no differences on brain activation were found in LMI when the right or left body side was stimulated. In a previous study of our lab [6], we have shown that CP children and adults elicited higher SEP amplitudes in contralateral hemisphere when left dominant side of motor impairment was stimulated as compared to right dominant side of motor impairment. Thus, it seems that CP individuals with left- and right-dominant motor impairments differ on brain processing of lateralized bodily information. An unusual pattern of bilateral cortical activation and recruitment of ipsilateral tracts have been usually linked to widespread cortical reorganization after brain lesions [41-44]. Moreover, our findings are in agreement with previous studies showing that motor impairments are different depending on the paretic dominant side of motor impairment after unilateral CP lesions. Thus, for instance, Van Kampen and colleagues [45] reported that children with left hemiparesis had longer decision time when asked to intercept a ball located 4 meters away and started their reach movement earlier than healthy controls and children with right hemiparesis. In addition, Craje and colleagues [46] observed that participants with right hemiparesis had more difficulties in switching between different grip types than participants with left hemiparesis. Our findings are also in agreement with previous data showing that bilaterally impaired CP children with spastic diplegia displayed higher intrahemispheric coherence for delta, beta and theta EEG bands in the left than in the right hemisphere [47]. In our opinion, all these results support the view that brain damage to right or to left hemisphere may have led to different plasticity mechanisms in cerebral palsy. In the present study, we observed that CP individuals with RMI displayed an asymmetrical pattern of brain activity more similar to that exhibited by healthy controls [48] than that of individuals with LMI. One possible explanation for these differences could be that damage of the left hemisphere releases the right hemisphere from its non-dominant role, while damage of the right hemisphere only emphasizes the usual role of the left hemisphere. Thus, for example, due to the dominant role of the left somatosensory cortex in sensorimotor integration for complex finger movements [49], damage of this hemisphere may have lead to a contralateral directed plasticity phenomenon in CP individuals with RMI. Nevertheless, further research should be necessary to elucidate the role of hemispheric dominance on somatosensory processing in CP individuals and the potential mechanisms of this differentiation.

Our study has some limitations, which should be taken into account for the interpretation of the results. Firstly, although our sample of persons with CP seems to be representative of a large population in the community, sample was small and heterogeneous. Thus, it could be that our sample size was not sufficient to reach an appropriate statistical power and to show differences between individuals with LMI and RMI on behavioral measures of somatosensory processing. In addition, the wide range of age, cognitive levels and underlying brain lesions as well as the presence of different subtypes of CP, have also limited the conclusions of the study. Moreover, it is possible that CP individuals may have been developing a left hemispheric dominance before their lesion. Nevertheless, the early onset of this pathology (in most cases, with a prenatal beginning) minimizes the influence of a possible left-hemispheric dominance development in comparison with several years of brain reorganization in the childhood and adolescence. On the other hand, the study should be replicated in hemiparetic CP individuals with clear and limited brain lesions. Finally, somatosensory-evoked potentials provide information from brain functioning arising from sensory cortices and, therefore, the influence of subcortical brain structures in somatosensory processing remains unexplored. Nevertheless, our study lays the scientific basis for implementation of further research on a scarcely investigated topic.

## Conclusion

Our findings suggest a different somatosensory cortical organization in participants with CP associated with asymmetrical motor impairments. Given that activity-dependent plasticity plays a key role in the evolution of clinical signs linked to motor dysfunctions in CP [42] and that training and rehabilitation interventions that target these maladaptive brain changes have already shown beneficial effects in several sensory and motor disorders [50], further research must elucidate the mechanisms of plastic changes associated to motor impairments in CP and relate them to specific rehabilitation interventions for these individuals.

## Competing interests

The authors declare that they have no competing interests.

## Authors’ contributions

IR carried out the recruitment of subjects, EEG recordings and somatosensory assessment. IR and PM conceived of the study, and participated in its design and coordination. IC, IP and AG participated in the design of the study and performed the statistical analyses. PM, IR, IC and IP prepared a draft of the manuscript. All authors read and approved the final manuscript.
